# One-Step Biofunctionalization of Quantum Dots with Chitosan and *N*-palmitoyl Chitosan for Potential Biomedical Applications

**DOI:** 10.3390/molecules18066550

**Published:** 2013-06-04

**Authors:** Joyce C. C. Santos, Alexandra A. P. Mansur, Herman S. Mansur

**Affiliations:** Center of Nanoscience, Nanotechnology and Innovation-CeNano^2^I, Department of Metallurgical and Materials Engineering, Federal University of Minas Gerais, Av. Antônio Carlos, 6627 – Escola de Engenharia – Bloco 2 – sala 2233, Pampulha, Belo Horizonte/MG, 31.270-901, Brazil; E-Mails: joycecruzsantos@yahoo.com.br (J.C.C.S.); aapmansur@hotmail.com (A.A.P.M.)

**Keywords:** carbohydrate, chitosan, glycoconjugate, quantum dot, biopolymer, bioconjugates

## Abstract

Carbohydrates and derivatives (such as glycolipids, glycoproteins) are of critical importance for cell structure, metabolism and functions. The effects of carbohydrate and lipid metabolic imbalances most often cause health disorders and diseases. In this study, new carbohydrate-based nanobioconjugates were designed and synthesized at room temperature using a single-step aqueous route combining chitosan and acyl-modified chitosan with fluorescent inorganic nanoparticles. *N*-palmitoyl chitosan (C-Pal) was prepared aiming at altering the lipophilic behavior of chitosan (CHI), but also retaining its reasonable water solubility for potential biomedical applications. CHI and C-Pal were used for producing biofunctionalized CdS quantum dots (QDs) as colloidal water dispersions. Fourier transform infrared spectroscopy (FTIR), thermal analysis (TG/DSC), surface contact angle (SCA), and degree of swelling (DS) in phosphate buffer were used to characterize the carbohydrates. Additionally, UV-Visible spectroscopy (UV-Vis), photoluminescence spectroscopy (PL), dynamic light scattering (DLS), scanning and transmission electron microscopy (SEM/TEM) were used to evaluate the precursors and nanobioconjugates produced. The FTIR spectra associated with the thermal analysis results have undoubtedly indicated the presence of *N*-palmitoyl groups “grafted” to the chitosan chain (C-Pal) which significantly altered its behavior towards water swelling and surface contact angle as compared to the unmodified chitosan. Furthermore, the results have evidenced that both CHI and C-Pal performed as capping ligands on nucleating and stabilizing colloidal CdS QDs with estimated average size below 3.5 nm and fluorescent activity in the visible range of the spectra. Therefore, an innovative “one-step” process was developed via room temperature aqueous colloidal chemistry for producing biofunctionalized quantum dots using water soluble carbohydrates tailored with amphiphilic behavior offering potential applications as fluorescent biomarkers in the investigation of glycoconjugates for the nutrition, biology, pharmaceutical, and medicine fields.

## 1. Introduction

Glycoconjugates such as glycoproteins and glycolipids have structures in which proteins or lipids are conjugated with saccharide moieties, and are crucial components of biological organisms, including cells, tissues of animals and plants. However, the exact roles of these glycoconjugates are not clearly understood due to the large number and complexity of the metabolic reactions involved, far beyond the simplified assumption that saccharides might play a role only in maintaining the structural properties of bioconjugates based on proteins and lipids [1,2,3]. Therefore, it is of paramount importance to advance the current knowledge in carbohydrate science and research using nano-technology as a powerful tool. Chitosan [poly-β(1→4)-2-amino-2-deoxy-d-glucose] is one of the most abundant polysaccharides available semi-processed from natural sources which has been used in a wide range of applications, such as pharmaceuticals, drug carrier and delivery, biomaterials, antimicrobial films, edible films in food packaging, and many others [4,5,6,7,8,9,10]. Chitosan is mainly produced from the alkaline deacetylation of natural chitin forming a copolymer composed of *N*-acetyl-d-glucosamine and d-glucosamine units available in different grades depending upon the content of acetylated moieties. The degree of deacetylation (DD) and the molar mass (MM) of the chitosan influence most of its properties, like solubility in water, mechanical behavior, optical transparency, chemical stability, biodegradability and others. Commonly, pure chitosan exhibits biodegradability and biocompatibility, but low solubility in aqueous media at physiological pH, leading to the formation of brittle films with unsuitable mechanical properties. For that reason, it has been blended with other polymers [11,12], crosslinked or grafted with chemical functionalities offering countless alternatives for developing new materials with a set of characteristics tailored for specific biomedical application.

In recent years, a new field of research drawing the attention of scientists has emerged in nanotechnology, based on building bioconjugates and hybrid nanomaterials, especially by combining organic and inorganic components in novel applications for *in vitro* and *in vivo* diagnostics and imaging, targeted therapeutics, tissue engineering and biosensing [13,14,15,16,17]. Thus, the combination of low dimension inorganic materials such as nanotubes, nanowires, nanorods, and quantum dots with organic molecules like synthetic polymers, carbohydrates, proteins and dendrimers offers a toolbox with innumerous alternatives for studying and investigating complex biological events and phenomena occurring in living organisms [18,19,20]. Quantum dots (QDs) are a new class of semiconductor fluorophores, which are actively researched for applications in health sciences. For instance, these luminescent nanocrystals may be used for investigating at a molecular level carbohydrate-mediated interactions such as carbohydrate-protein, carbohydrate-lipid and carbohydrate-carbohydrate relations occurring in biological processes [16,17,21]. However, to be used in biological environments they must exhibit compatibility with the physiological medium where water is abundant and with the large number of natural macromolecules. Therefore, surface chemical engineering of QDs is mandatory to render them water soluble and biocompatible. Additionally, the “ideal” surface of the designed nano-hydrid material would require an amphiphilic behavior by the presence of both hydrophobic and hydrophilic functionalities for interacting with a broad range of biological molecules at the interfaces. Thus, QDs have been produced with amphiphilic polymers allowing hydrophobic interactions, while the hydrophilic part provides water dispersibility. These properties arise from the inter- or intramolecular interactions among hydrophobic groups providing hydrophobic micro-nanodomains in aqueous solution [22]. Nonetheless, surface functionalized QDs must display suitable “hydrodynamic diameter” (H_D_) to be useful as fluorescent probe for most biological application, due to the overall structural size limitations. In that sense, the development of innovative procedures for producing biofunctionalized QDs, with chemical stability, narrow size distributions, and biocompatibility, associated with the least possible H_D_ is a very promising theme for research. Unexpectedly, despite the wide range of potential applications, only a few reports have been published combining quantum dots with chitosan and derivatives and the majority have focused on films and nanoparticles of chitosan with quantum dots embedded in the polymer matrix [23,24]. No report has been found in the literature using chitosan and *N*-palmitoyl chitosan as direct capping ligands for producing QDs in colloidal water dispersions.

Thus, in this study, a novel class of fluorescent labeled glycoconjugates based on quantum dots capped by chitosan and *N*-palmitoyl chitosan in aqueous media is presented. These biofunctionalized nanocrystals with amphiphilic biopolymer surfaces are considered promising tools for potential use as lipophilic dietary supplements in nutrition, pharmaceutics, and nanomedicine.

## 2. Experimental

### 2.1. Materials

All reagents and precursors, cadmium (II) perchlorate hexahydrate [Cd(ClO_4_)_2_·6H_2_O, Sigma, St. Louis, MO, USA], sodium sulfide nonahydrate (Na_2_S·9H_2_O, Synth, Belo Horizonte-MG, Brazil, >98%), sodium hydroxide (NaOH °99%, Merck, Darmstadt, Germany), acetic acid (CH_3_COOH °99.7%, Synth), ammonium hydroxide (NH_4_OH, NH_3_: 28%–30%, Merck) palmitic acid [IUPAC: hexadecanoic acid, CH_3_(CH_2_)_14_COOH), °99%, Sigma, St. Louis, MO, USA), 1-ethyl-3-[3-dimethylaminopropyl]-carbodiimide hydrochloride (C8H17N3·HCl, °98%, EDC, Sigma), *N*-hydroxysulfosuccinimide sodium salt (C_4_H_4_NNaO_6_S, °98%, sulfo-NHS, Sigma), methanol (CH_3_OH, 99.8%, Synth), and acetone (propanone, CH_3_COCH_3_, 99.8%, Synth) were used as received. Chitosan powder (Sigma, molar mass, MM = 310,000 to >375,000 g/mol, degree of deacetylation, DD ≥ 75.0%, and viscosity 800–2,000 cPoise, at 1% in 1% acetic acid) was used as the reference ligand. De-ionized water (DI-water, Millipore Simplicity^™^) with resistivity of 18 MΩ·cm was used in the preparation of all solutions. All preparations and synthesis were performed at room temperature (23 ± 2 °C) unless specified.

### 2.2. Preparation Methods of CdS Precursor Solutions

Approximately 0.196 g of Na_2_S·9H_2_O was added to DI-water (75 mL) in a 100 mL flask and homogenized under moderate manual stirring for 10–15 min at room temperature. Then, the volume was completed to 100 mL with DI-water. This sulfur precursor stock solution (8 × 10^−3^ mol L^−1^) was referred to as “SOL_S”.

Cd(ClO_4_)_2_·6H_2_O (approximately 0.4193 g) was added to DI-water (75 mL) in a 100 mL flask and homogenized under moderate manual stirring for 10–15 min at room temperature. Then, the volume was completed to 100 mL with DI-water. This cadmium precursor stock solution (1 × 10^−2^ mol L^−1^) was referred to as “SOL_Cd”.

### 2.3. Preparation of Reference Chitosan (CHI) 1.0 % (w/v) Solution

Chitosan solution (1%, w/v) was prepared by dispersing chitosan (2.59 g) in an aqueous solution (2%, v/v) of acetic acid (250 mL). The dispersion was placed under constant stirring overnight at room temperature, until complete solubilization has occurred. The solution of chitosan was diluted for nanoparticle synthesis at 0.45 g L^−1^ in water (referred to as “SOL_CHI”).

### 2.4. Procedure for Preparation of N-Palmitoyl Chitosan (C-Pal)

The palmitic acid was conjugated to the chitosan polymer using 1-ethyl-3-[3-dimethylamino-propyl]carbodiimide hydrochloride (EDC) as a “zero-length” crosslinking agent in the presence *N*-hydroxysulfosuccinimide sodium salt (sulfo-NHS). EDC in the presence of sulfo-NHS converts the carboxyl groups of palmitic acid to amine-reactive esters [17,25]. These esters commonly react with available amine groups in chitosan, forming a conjugate of CHI and palmitic acid linked by stable amide bonds [RC(O)NR’R’’] referred to as *N*-palmitoyl chitosan (C-Pal).

To prepare *N*-palmitoyl chitosan, chitosan (CHI, 1.0 g) was dissolved in aqueous solution (2%, v/v) of acetic acid (100 mL) under constant stirring overnight at room temperature (23 ± 2 °C). Then, after complete solubilization, methanol (85 mL) was added. In the sequence, palmitic acid powder (fatty acid, 0.02 g) and EDC and sulfo-NHS solution (15 mL, 0.07 mol L^−1^ in methanol) were added to the system. The molar ratio EDC:palmitic acid was 1:1. The reaction occurred under moderate stirring for 24 h at room temperature. The *N*-palmitoyl chitosan product was collected by precipitation by adding methanol-ammonia solution (200 mL, 7:3 v/v). After washing (five times with DI water, methanol and acetone) and filtering, it was dried in an oven for 48 h at 40 ± 2 °C. 

C-Pal solution for quantum dot synthesis (1.0%, w/v) was prepared by dissolving C-Pal flakes (0.5 g) in acetic acid aqueous solution (50 mL, 2%, v/v) under moderate magnetic stirring overnight until complete solubilization has occurred. The solution of C-Pal was diluted for nanoparticle synthesis to 0.45 g L^−1^ in water (referred to as “SOL_C-Pal”).

### 2.5. Synthesis of CdS Nanoparticles in CHI and C-Pal Solutions

**Figure 1:**
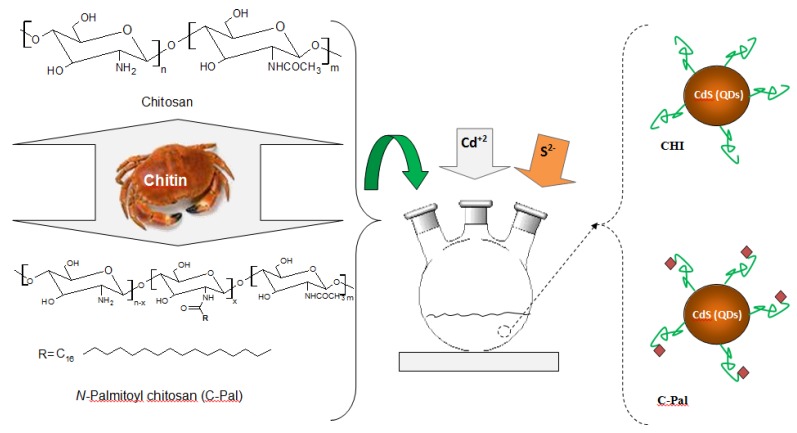
Schematic representation of the designed experimental procedure for the CdS-carbohydrates colloidal systems; Molecular structures of chitosan and *N*-palmitoyl chitosan (C-Pal).

CdS nanoparticles were synthesized *via* an aqueous route in a reaction flask using the stock solutions as detailed in the previous sections, Cd^2+^ and S^2−^ precursors, and CHI or C-Pal as capping ligands. A typical synthesis was carried out as follows: “SOL_CHI” or “SOL_C-Pal” (47 mL, pH = 3.8 ± 0.1) were added to the reaction vessel. Under moderate magnetic stirring, cadmium precursor solution (4.0 mL, Cd^2+^, “SOL-Cd) and sulfur source solution (2.5 mL, S^2−^, “SOL_S”) were added to the flask (S^2−^:Cd^2+^ molar ratio was kept at 1:2). The solution turned yellowish and sampling aliquots of 3.0 mL were collected at different time intervals (after preparation, 1 day, and 4 days) for UV-vis spectroscopy measurements that were used for kinetics analysis and colloidal stability evaluation. After 4 days no changes were detected which was considered as colloidal stability of the system. A schematic representation of the experimental procedure performed for the synthesis of CdS_CHI and CdS_C-Pal bioconjugate systems is shown in Figure 1.

### 2.6. Characterization of Carbohydrates

Chitosan and C-Pal powder/flakes, solutions (“SOL_CHI” or “SOL_C-Pal”) or films were used for carbohydrate characterization. For the preparation of films, polymer solutions (“SOL_CHI” and “SOL_C-Pal”) were poured into plastic molds (Petri dish, polyethylene, round-plate shape, diameter = 85 mm, height = 10 mm) and allowed to dry for 48 h at 23 ± 2 °C in a desiccator followed by additional 48 h in an oven at 40 ± 2 °C.

#### 2.6.1. Fourier Transform Infrared Spectroscopy (FTIR)

Fourier transform infrared spectroscopy (FTIR) was used to characterize the presence of specific chemical groups in chitosan and *N*-palmitoyl chitosan derivative. FTIR was performed over the range of 650–4000 cm^−1^ (Nicolet 6700, Thermo Fisher, Waltham, MA, USA) using the attenuated total reflectance spectroscopy method (ATR). Chitosan and functionalized chitosan derivative (C-Pal) flakes were placed on the ATR crystal prism (ZnSe), and 16 scans were acquired at 2 cm^−1^ resolution with background subtraction.

#### 2.6.2. Thermal Analysis (TG/DSC)

Thermogravimetric (TG) and Differential Scanning Calorimetry (DSC) analyses were performed using SDT Q-600 simultaneous TGA/DSC instrument (TA Instruments, New Castle, DE, USA). Samples of about 4.0 ± 1.0 mg were used for the experiments at a heating rate of 10 °C min^−1^ up to 400 °C. The samples were loaded without pressing into an open aluminum crucible. The TG, Derivative Thermogravimetric Analysis (DTG) and DSC curves were recorded simultaneously with 0.1 μg sensitivity. The analysis was performed under continuous flow of dry nitrogen gas (100 mL min^−1^).

#### 2.6.3. Degree of Swelling (DS) in Phosphate Buffer (PBS)

For fluid-uptake measurements, squared specimens (10 mm × 10 mm) cut from CHI and C-Pal films were weighed (W_o_) before being immersed in PBS (Ph = 7.4) at room temperature (controlled at 23 ± 2 °C). After immersion for different time periods (0.5, 2.0, and 24 h), the samples were carefully removed from the medium and, after wiping off solution excess on the surface with filter paper, they were weighed for the determination of the swollen film weight (W_f_). The degree of swelling (DS) was calculated as indicated in equation (1) (average, n = 3):

DS (%) = (W_f_ − W_o_/W_o_) × 100% (1)

#### 2.6.4. Surface Contact Angle (SCA)

The influence of acylation with palmitic acid on the hydrophilic/hydrophobic behavior of chitosan was estimated via contact angle measurements that were carried out by depositing DI water droplets (50 μL) on chitosan and C-Pal films. The apparatus used for measurements was a DSC-W70 digital camera (Sony, Tokyo, Japan) with image analysis software.

#### 2.6.5. Qualitative and Scanning Electron Microscopy (SEM) analysis

Qualitative visual observations and microstructural evaluations were conducted on films. For the evaluation of films morphology, scanning electron secondary electrons (SE) images (SEM, JSM 6,360LV, JEOL/NORAN, Tokyo, Japan) were taken by using an accelerating voltage of 10–15 kV. Before examination, samples were coated with a thin gold film by sputtering using low deposition rate, cooling of substrate and maximum distance between target and sample in order to avoid sample damage.

### 2.7. Characterization of CdS Quantum Dots

#### 2.7.1. UV-Visible Spectroscopy (UV-Vis)

UV-Vis spectroscopy measurements were conducted using Perkin-Elmer equipment (Lambda EZ-210, Waltham, MA, USA), wavelength from 600 nm to 190 nm, in transmission mode, using quartz cuvette. The absorption spectra were used to monitor the reaction for the formation of CdS QDs and their relative colloidal stability in the medium. Moreover, based on the absorbance curves, it was possible to calculate the average nanoparticles sizes and their optical properties. All experiments were conducted in triplicates (n = 3) unless specifically noted. 

#### 2.7.2. Photoluminescence spectroscopy (PL)

Photoluminescence (PL) characterization of the CdS-bioconjugated nanohybrids was conducted based on spectra acquired at room temperature using a high power xenon light source (HPX-2000, Mikropack, Ostfildern, Germany) coupled to an Ocean Optics USB2000 VIS-NIR spectrophotometer. The relative activity was calculated by subtracting the background of the sample without QDs. All tests were performed using a minimum of three repetitions (n ≥ 3). Additionally, QD colloidal media were placed inside a “darkroom-chamber” where they were illuminated by a UV radiation emission bulb (λ_excitation_ = 245 nm, 6 W, Boitton Instrumentos, São Paulo, Brazil). Digital color images were collected when the QDs fluoresced in the visible range of the spectra. 

#### 2.7.3. Transmission Electron Microscopy (TEM)

Nanostructural characterizations of the QD-bioconjugates, based on the images and electron diffraction patterns (ED), were conducted using transmission electron microscopy (TEM, Tecnai G2-F20-FEI microscope, Hillsboro, OR, USA) at an accelerating voltage of 200 kV. For TEM analyses, samples were prepared by dropping the colloidal dispersion onto a porous carbon grid. 

#### 2.7.4. Dynamic Light Scattering (DLS) analysis

Dynamic Light Scattering (DLS) analyses were carried out using a Brookhaven ZetaPALS instrument (laser light of 660 nm, square acrylic cells of 4.5 Ml, Holtsville, NY, USA). For DLS analysis of quantum dots nanohybrids, the colloidal dispersions were filtered three times through a 0.45 μm aqueous syringe filter (Millex LCR 25 mm, Millipore, Ostfildern, Germany) to remove any dust. Then, they were placed into ultrasonic bath for approximately 10 s at the time of experiment. Samples were measured at 25.0 ± 2.0 °C and the light scattering was detected at 90°. Each run took about three minutes, and three measurements were taken for each system and averaged out.

## 3. Results and Discussion

### 3.1. Characterization of Carbohydrates

#### 3.1.1. Fourier Transform Infrared Spectroscopy (FTIR)

**Figure 2:**
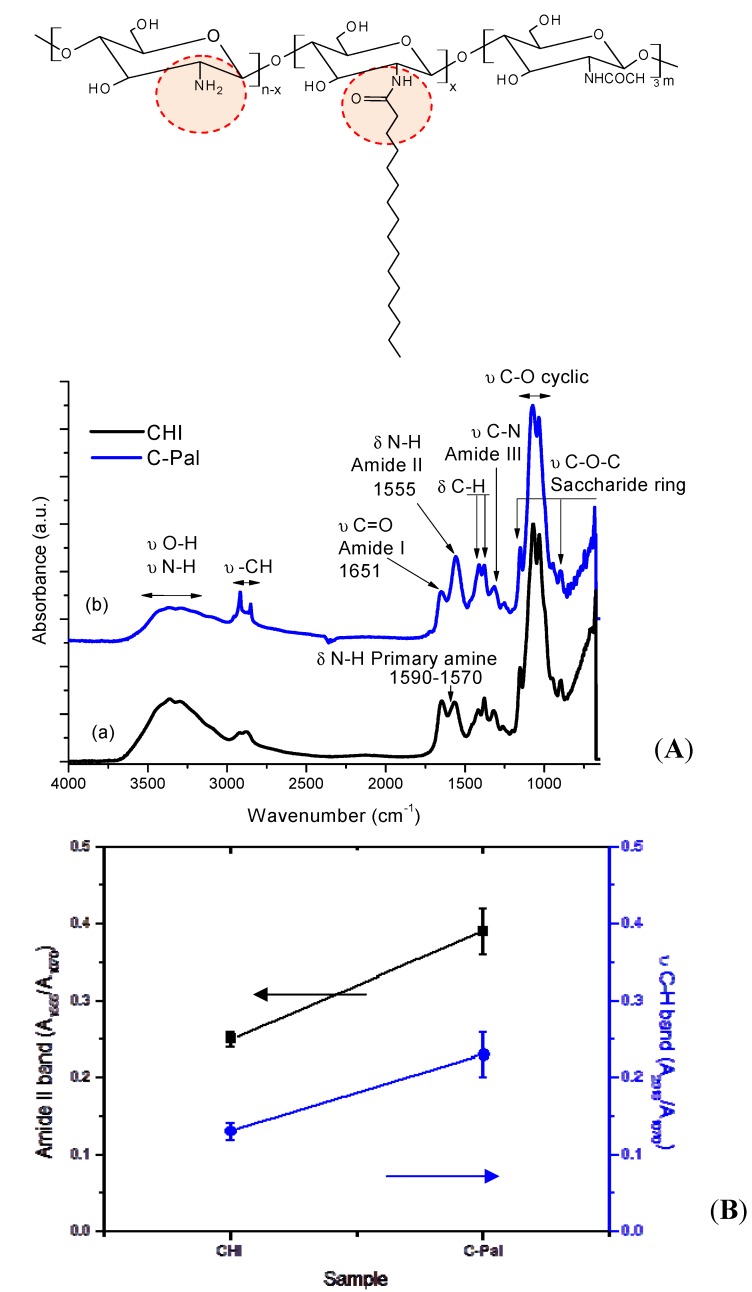
(**A**) Infrared spectra of (a) chitosan and (b) C-Pal. (**B**) Evolution of amide II band (squares) and ν C-H band (circles) due to *N*-acylation reaction.

The FTIR spectra of CHI and C-Pal are showed in Figure 2A. Since chitosan is a copolymer composed of *N*-acetyl-d-glucosamine and d-glucosamine repeating units, the absorption peaks at 1651 cm^−1^, 1590–1570 cm^−1^, and 1555 cm^−1^, assigned to carbonyl stretching of secondary amides (amide I band), N-H bending vibrations of the deacetylated primary amine (-NH_2_), and N-H bending vibrations of the amide II band, respectively, are presented in the starting material (CHI) [8,26,27,28]. The degree of deacetylation (DD) of chitosan was calculated from FTIR spectrum using Equation (2) [29]:

A_1320_/A_1420_ = 0.3822+ 0.03133 × (100−DD) (2)

where A_1320_ and A_1420_ are the absorbance associated with amide III C-N bonds and C-H symmetrical deformation vibrations, respectively. The obtained value was DD = 78.9 ± 0.1% in accordance with the manufacturer (≥75.0%).

The changes in the macromolecule of chitosan after the palmitic acid acylation reaction can be observed by infrared spectroscopy. After *N*-acylation [Figure 2A(b)], the vibrational band corresponding to primary amine groups (1570–1590 cm^−1^) is not apparent and the absorption band at 1555 cm^−1^, associated with the formation of new amide bonds due to acylation, increased. The same trend was observed for stretching C-H vibrations bands at 2918 cm^−1^ and 2848 cm^−1^ related to acyl chains [26,27,28]. Figure 2B shows the evolution of amide II (1555 cm^−1^) and νC-H (2918 cm^−1^) bands, using C-O stretching band at 1070 cm^−1^ as the internal reference band, as a result of palmitic acid grafting to chitosan chain [30]. The degree of *N*-acylation of glucosamine groups (DA) was also evaluated by FTIR from the ratio of absorbances at 1655 cm^−1^ (A_1655_, amide I vibration) and at 3450 cm^−1^ (A_3450_, hydroxyl band) using equation (3) [31]. Thus, the degree of substitution of C-Pal sample was estimated to be 20 ± 3%:

DA (%) = [(A_1655_/A_3450_)_C-Pal_ − (A_1655_/A_3450_)_CHI_] × 100 (3)

#### 3.1.2. Thermal Analysis (TG/DSC)

**Figure 3:**
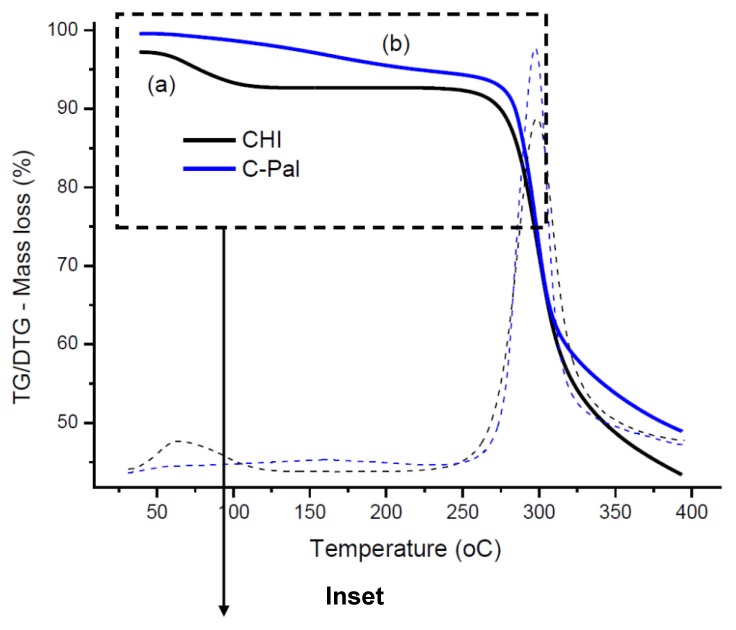

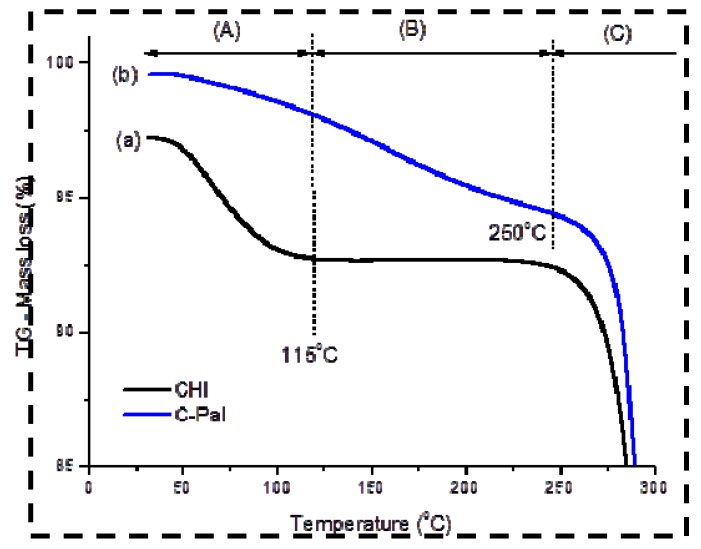
TG/DTG curves of (**a**) chitosan and (**b**) C-Pal; (Inset: temperature zones of thermal events of chitosan/C-Pal).

**Figure 4:**
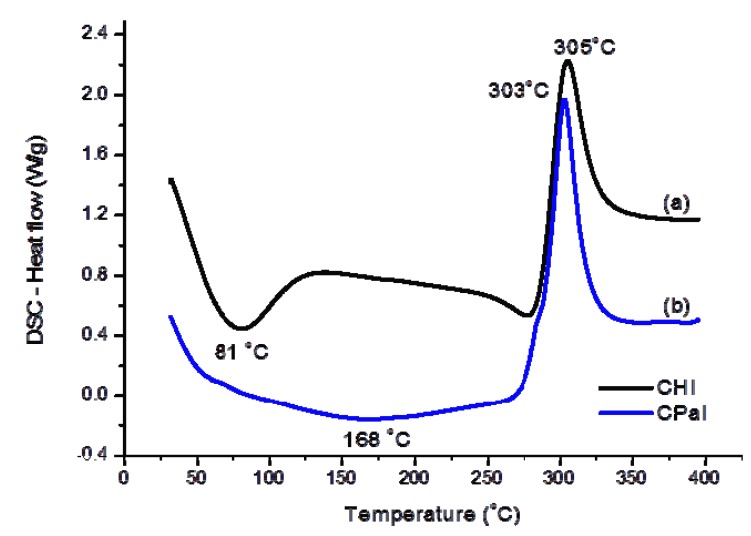
DSC curves of (**a**) chitosan and (**b**) C-Pal.

Figure 3 (TG/DTG) and Figure 4 (DSC) illustrate the thermal analyses of chitosan and chitosan derivative. Two major steps of mass loss with temperature can be observed in the TG/DTG curve of chitosan (Figure 3a). The first region ranges from 30 °C to 115 °C (inset of Figure 3) with a mass loss of about 4.6%, which is due to the evaporation of water physically adsorbed and absorbed in the inner polymeric network. This event may be associated with a broad endothermic peak centered at about 81 °C in DSC curve (Figure 4a). 

The second region starts at approximately T = 225 °C and the corresponding mass loss of about 49% is attributed to the thermal degradation of the polymeric chain [32,33]. Figure 3b shows that the C-Pal derivative also had two distinct zones of mass loss. The first stage, beginning at 30 °C and ending at about 250 °C, overlaps the endothermic events (Figure 4b) of water evaporation [Zone (A) in Figure 3 inset] and decomposition of palmitoyl group attached to the chitosan [Zone (B) in Figure 3 inset], assuming as the reference the decomposition of fatty acid (not shown) beginning at 140 °C. The total mass loss in this step was 4.7%. Considering that water removal occurred from 30 to 115 °C, the moisture content of C-Pal was about 2%, which is smaller than the value measured for chitosan. That could be assigned to the incorporation of palmitic groups in the chitosan backbone that reduced the relative concentration of hydrophilic groups in the polymer [34]. The second endothermic stage of chitosan derivative (mass loss of 46%) started at about 250 °C [Zone (C) in Figure 3 inset] and is associated with the degradation of chitosan. Also, from DSC curves (Figure 4), the decomposition peak of C-Pal (at 303 °C) occurred at lower temperature than chitosan (at 305 °C). So, it may be considered that the introduction of the (16-carbons) palmitoyl group has influenced the formation of hydrogen bonds and other hydrophilic interactions in the chitosan network disrupting the semi-crystalline structure of the polymer [33,35]. 

#### 3.1.3. Degree of Swelling (DS) in Phosphate Buffer (PBS)

**Figure 5:**
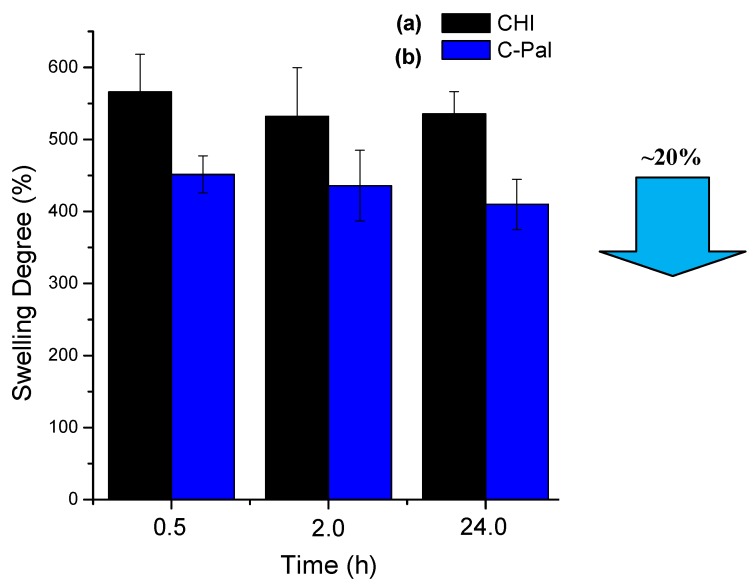
Degree of swelling of chitosan (**a**) and palmitoyl-chitosan (**b**) films.

The results of the degree of swelling for chitosan and *N*-palmitoyl-chitosan films in PBS are shown in Figure 5. It can be observed that the systems have reached the equilibrium after 24 h of swelling assay (slight decrease in DS within the statistical experimental error). However, the chemical modification of the chitosan backbone by grafting the alcyl group (IUPAC: alkanoyl, R-CO-) has significantly affected the average DS values, from DS = 550% of the pure chitosan film to DS = 450% of C-PAL films (a relative reduction of 20% in the DS values). That could be attributed to the hydrophobic behavior of the palmitoyl groups that have partially reacted with the amines from the chitosan, leading to the reduction of the amount of water uptake by the hydrogel network. So, as a hydrogel, the overall hydrophilicity/hydrophobicity of the chitosan polymeric network is mostly caused by the presence of chemical groups such as hydroxyls, amides, amines and others that can be found within the polymer backbone or as lateral chains [9]. 

#### 3.1.4. Surface Contact Angle (SCA)

**Figure 6:**
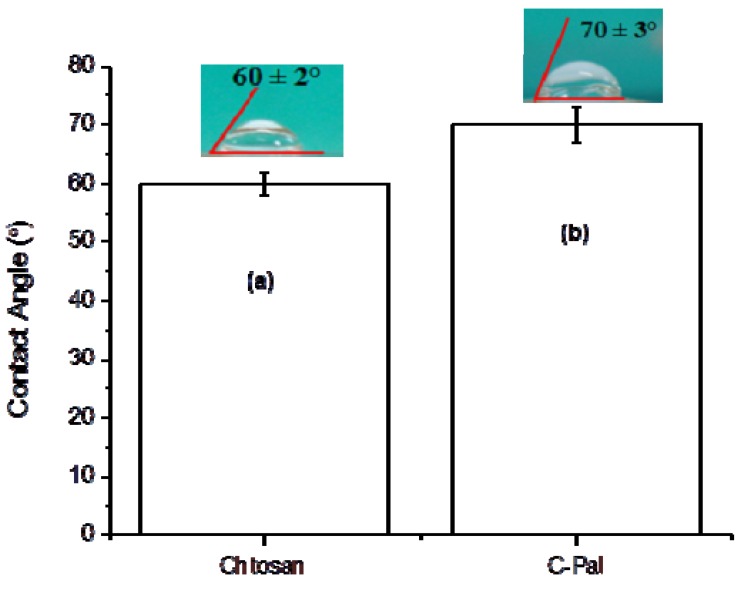
Contact angle measurements of chitosan (**a**) and palmitoyl-chitosan (**b**) films.

A hydrogel is defined as a highly hydrated three-dimensional hydrophilic polymer network. However, the high level of hydrophilicity of the polymer molecules required to form the hydrogel cannot be directly associated with very low water contact angle. On the contrary, the surface of a hydrogel often shows a remarkably high water contact angle at the hydrogel-air interface [36]. In the present study, the contact angle was evaluated using water droplets as a qualitatively method for comparing the relative variation of hydrophobic/hydrophilic behavior of the chitosan films and the results are presented in Figure 6. It may be noted that the acylation of the chitosan has increased the average contact angle by approximately 16% (or ∆θ = 10°), from 60 ± 2° of pure chitosan to 70 ± 3° of C-Pal. That could be attributed to the presence of more hydrophobic groups in the C-Pal films compared to chitosan, assuming that the polymer has the capability of altering the surface configuration by means of rotational and rearrangement of functional groups, towards reaching the equilibrium minimizing the surface energy at the interfaces. 

#### 3.1.5. Qualitative and Scanning Electron Microscopy (SEM) Analysis

**Figure 7:**
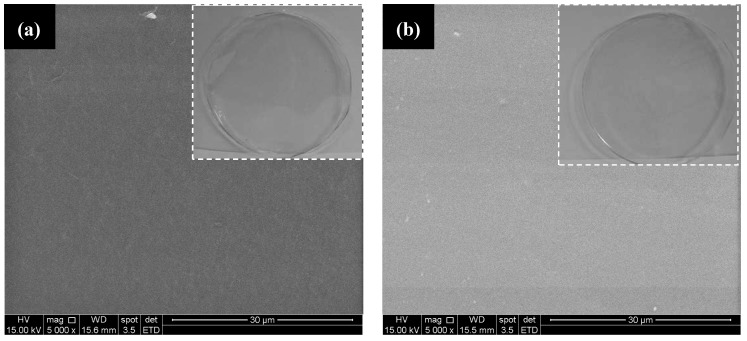
SEM images of chitosan (**a**) and palmitoyl-chitosan (**b**) films; (Inset: digital images with no magnification).

Typical SEM images of chitosan and *N*-palmitoyl chitosan films are shown in Figure 7a,b, respectively. Qualitatively, the SEM images indicated the formation of very homogenous films for both systems without any detectable voids or other heterogeneities even at 5,000× magnification (with minor difference in contrast). Also, similar characteristics can be observed in the images captured by digital camera (Sony^®^, Cybershot^®^, 12 Mpixels) without magnification (inset Figure 7). It means that chitosan and chemically modified chitosan have formed fairly uniform and optically transparent films using a simple solution casting and evaporation methods.

### 3.2. Characterization of CdS Quantum Dots

#### 3.2.1. UV-Visible Spectroscopy (UV-Vis)

Some background on physics and chemistry of semiconductor nanocrystals is needed to characterize the bioconjugates. Essentially, due to their ultra-small dimension, semiconductor nanoparticles will present a “quantum-confinement effect”, related to the exciton (or “hole-electron” pair) generated by exciting radiation [37]. That means, the nanocrystals after reaching a specific threshold in particle size (R = radius), they are referred to as quantum dots with broader energy of band gap (E_QD_) than the original bulk material [20]. In this study, the average nanoparticle size in colloidal suspension was determined from Henglein’s empirical model [38] which relates the CdS nanoparticle diameter (2R) to the optical “excitonic absorption” (λ_exc_) from UV-vis spectra. This procedure has been widely utilized for estimating the size of semiconductor nanoparticles directly *in situ* from colloidal dispersions *via* the UV-vis spectroscopy method. 

**Figure 8:**
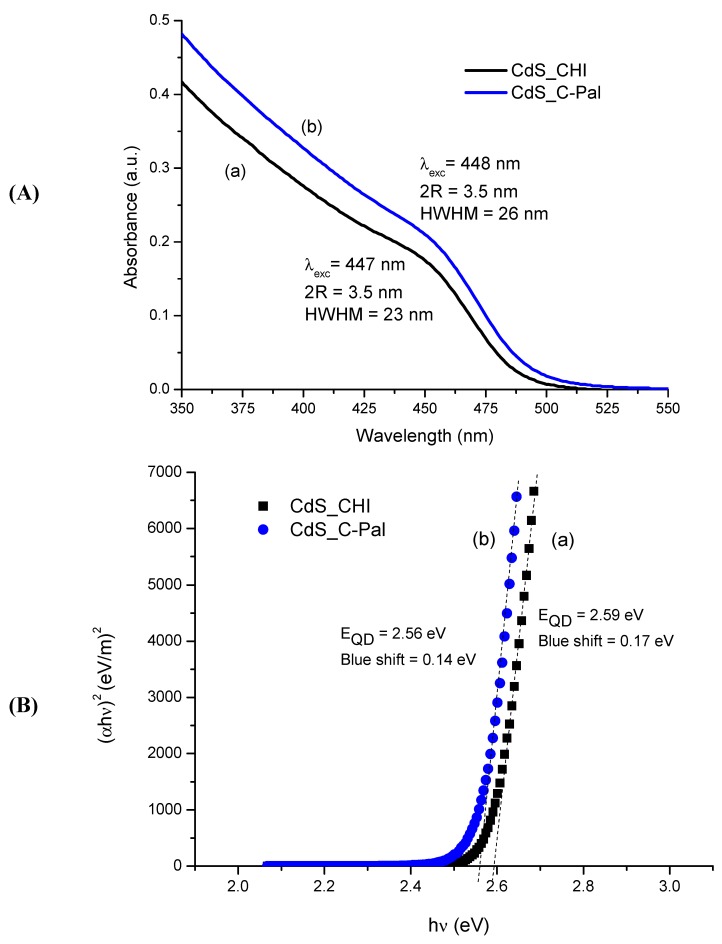
(**A**) UV-visible absorption spectra and (**B**) optical absorption spectra of CdS nanoparticles in carbohydrates media after four days preparation: (a) chitosan; (b) *N*-palmitoyl chitosan (C-Pal).

In Figure 8A the UV-vis spectroscopy results of the semiconductor nanoparticle colloids in water media using the carbohydrates as the stabilizing ligands are presented. The CdS nanocrystals were nucleated and stabilized with the carbohydrate-based ligands (after four days) with equivalent average sizes of 3.5 nm (2R) for both chitosan and *N*-palmitoyl chitosan, that were estimated from Henglein’s empirical model and wavelength value at the first excitonic transition (λ_exc_, nm), using Equation (4):

2R_CdS_ = 0.1/(0.1338 − 0.0002345 λ_exc_) (4)

The UV-vis spectroscopy method may also be used to estimate the band gap shift (“blue-shift”) caused by the “quantum-size effect” in the semiconductor nanoparticles. The optical band gap (E_QD_) was assessed using the “Tauc relation” [39] for obtaining the wavelength value (λ_onsetc_) associated with the “absorbance onset”, as showed in Equation (5):

(αhν)^2^ = B(hν − E_QD_) (5)

where α is the absorption coefficient, hν is the photon energy, B is the band form parameter, E_QD_ is the optical band gap of the nanoparticles.

Therefore, one can estimate the direct band gap value from the plots of (αhν)^2^ versus (hν) and extrapolating the straight portion of the graph to (hν) axis, *i.e.*, at α = 0 (dashed lines in Figure 8B). It can be observed very similar values of the optical band gap, E_QD_ = 2.59 ± 0.02 eV and 2.56 ± 0.02 eV, calculated for CdS colloidal suspensions using CHI and C-Pal, respectively. As these values are higher than the “bulk value” of 2.4 eV for CdS [20], it may be affirmed that the CdS quantum dots were effectively synthesized through this single-step route using chitosan and its acyl-derivative as the capping moieties. The results extracted from UV-Visible spectra and optical absorbance analyses are summarized in Table 1. 

**Table 1:** Quantum dot parameters: Band-gap energy, blue-shift, and estimated particle size.

System	Parameters	Values after 5 days
CdS-CHI	Band Gap (eV)	2.59 ± 0.02
	Blue Shift (eV)	0.17 ± 0.02
	λ_exc_ (nm)	447 ± 2
	2R (nm)	3.5 ± 0.1
	HWHM (nm)	23 ± 1
CdS-CPal	Band Gap (eV)	2.56 ± 0.02
	Blue Shift (eV)	0.14 ± 0.02
	λ_exc_ (nm)	448 ± 2
	2R (nm)	3.5 ± 0.1
	HWHM (nm)	26 ± 1

**Figure 9:**
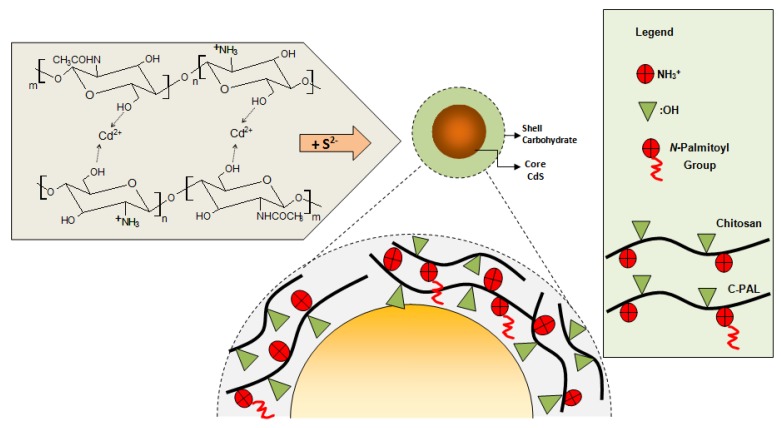
Schematic representation of the mechanism of interactions between the CdS quantum dots and the chemical groups from the carbohydrate chains.

The half-width at half-maximum (HWHM) on the low energy side of the first exciton absorption peak position can be used as a relative indicative of the size distribution of nanoparticles [40], with smaller HWHM corresponding to narrower size distribution [41]. Usually, the HWHM indexes of nanocrystals synthesized using organometallic precursors are in the range from 10 nm to 20 nm [40,42], compared to QDs prepared in aqueous solutions that have a broader peak width [43]. In this study, similar HWHM values were estimated for both biofunctionalized conjugates CdS_CHI and CdS_C-PAL, 23 ± 1 nm and 26 ± 1 nm, respectively. It can be seen, as expected, that both parameters were broader than those values typically reported for CdS prepared using non-aqueous routes. Besides, despite statistically similar HWHM values for both systems, the *N*-palmitoyl chitosan glycoconjugate seems to have slightly increased the dispersion of the QDs distribution, but it has showed equivalent average size compared to chitosan. It could be suggested that the acyl groups grafted to the chitosan chain (C-Pal) had changed the balance of hydrophilic/hydrophobic interactions between the chemical groups of the carbohydrate chain and also the electrostatic interactions of charged groups with the aqueous solvent of the colloidal suspension. Additionally, steric hindrance of the *N*-palmitoyl group may have affected the capping behavior of the glycoconjugate towards the formation of QDs. However, the reaction of Cd^2+^ with S^2−^ forming CdS crystals is very favorable under the thermodynamic (Gibbs free energy change, ΔG < 0) and kinetic (“solubility product constant”, K_sp_= 8.0 × 10^−27^) conditions used for the synthesis [44,45]. Thus, the overall effect of chemically modifying the chitosan with acyl groups has not significantly changed the average size and distribution of CdS quantum dots. Consequently, in the present study, the results have clearly evidenced that chitosan and *N*-palmitoyl chitosan were remarkably effective as ligands for stabilizing colloidal semiconductor quantum dots in aqueous media. It is plausible to consider that the most probable mechanism acting on the system is the reduction of the very high surface energy of the CdS QDs by the interactions of the chemical functionalities from the carbohydrate chains, as schematically represented in Figure 9. 

Owing to the “excess” of cadmium ions compared to sulfides in the synthesis, [Cd^2+^]/[S^2−^] = 2:1, it is considered that the stabilization of bioconjugates occurred predominately because chitosan is a multi-nucleophilic polymer with *N*-amino and hydroxyl functional groups. Regarding the acylated chitosan (C-Pal), a similar behavior is expected to take place considering that the nucleophilic amino groups are readily protonated (positively charged) in acid medium, as the pKa of chitosan in water is approximately 6.5 [46]. *N*-acylation of chitosan is favored compared to *O*-acylation because amines are more nucleophilic than hydroxyls (carbons 3 and 6 of the chitosan chain). Also amides are more stable molecules due to the effect of resonance localization of the lone pair electrons on nitrogen into the carbonyl “π” system [47]. That means, the presence of nucleophilic sites in the chitosan polymer was not significantly altered by grafting the *N*-palmitoyl moieties, leading to the formation of the lipid glycoconjugate (C-Pal). This assumption is supported by the findings described in the previous sections regarding the nucleation and stabilization of CdS QDs with very similar sizes prepared using chitosan and *N*-palmitoyl chitosan ligands in water media. It should be stressed that this is a simplified approach to the system, as many other interactions may also be relevant in the dynamic and complex process of synthesizing colloidal nano-hybrids structures. Several hydrophilic, hydrophobic, and electrostatic interactions, hydrogen bonding, steric hindrances, and spatial macromolecule conformations are expected to occur in the organic capping ligands (CHI and C-Pal) and also at the nanointerfaces with the inorganic semiconductor nanocrystals. However, a more in-depth investigation of the adsorption phenomenon and mechanism is beyond the scope of the present study which would need additional research of the entire balance of forces involved at the interface of quantum dot/polymer ligand [13,14,48].

#### 3.2.2. Photoluminescence Spectroscopy (PL)

Fluorescence spectroscopy was used to assess the surfaces and interactions at the quantum dot-ligand nanointerfaces in the water medium. PL behavior of quantum dot bioconjugates in aqueous colloids reflects the overall contributions occurring in the hybrid systems, dependent mostly on size of particles, self-organization, charges, capping ligand amount and chemistry, type of defects and depth of trap states.

**Figure 10:**
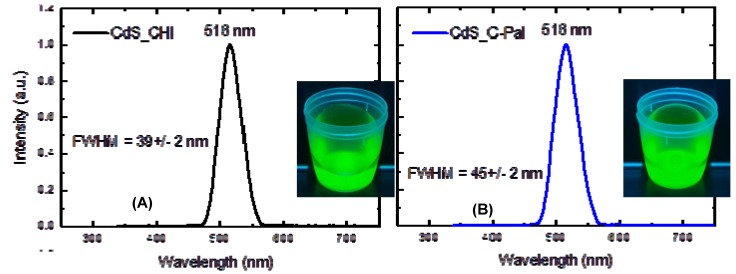
PL spectra obtained from (**A**) CdS-CHI and (**B**) CdS-C-Pal.

The fluorescence images and spectra of CdS nanoparticles stabilized with chitosan and *N*-palmitoyl chitosan are shown in Figure 10A,B, respectively. From the PL spectra it can be observed that in both systems the fluorescence is dominated by a green emission centered at about 516 nm. The digital images captured from the colloidal bioconjugates in aqueous media under UV excitation (“darkroom”, λ = 245 nm) also clearly revealed that the recombination is occurring by the emission of green light. According to the literature, this green emission is favored by the synthesis of the nanoparticles under the condition of excess of the metal that enters in the lattice at interstitial sites (Cd_i_) [49,50] or it is attributed to interstitial sulfur (S_i_) formed through substitutional doping of CdS by other anions [51]. The condition of cadmium excess is compatible to the systems, synthesized with the molar ratio of [Cd^2+^]/[S^2−^]= 2:1.

From the PL spectra the full-width at half-maximum (FWHM) parameter that can be used as convenient index of size distribution (smaller FWHM is an indicative of narrower distribution) was also calculated. FWHM calculated values were 39 ± 2 nm and 45 ± 2 nm for CdS_CHI and CdS_C-PAL, respectively. In this sense, the results indicated the slightly higher dispersion of the size distribution of quantum dots stabilized with C-Pal glycoconjugate as previously evaluated by absorbance curves.

#### 3.2.3. Transmission Electron Microscopy (TEM)

**Figure 11:**
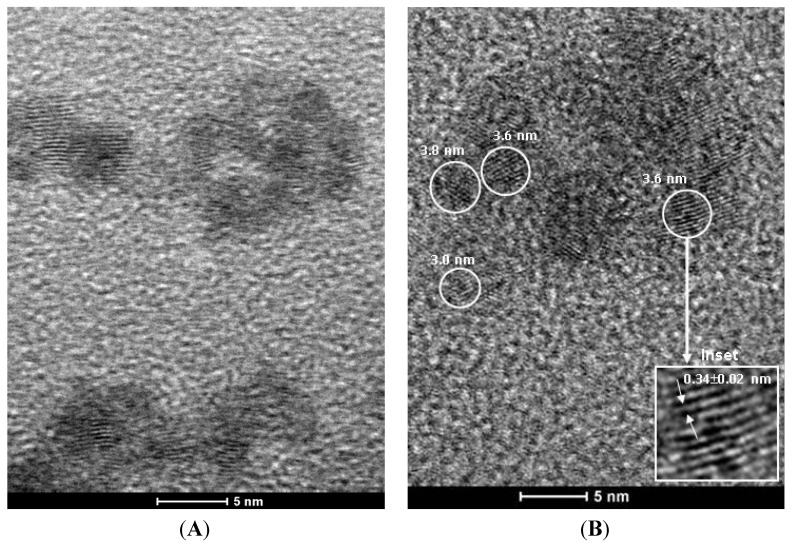
TEM image of CdS_CHI (**A**) and CdS_C-Pal (**B**); (inset: detailed nanocrystal plane spacing by electron diffraction and representative drawing).

In this study, biofunctionalized quantum dots were characterized using TEM for investigating the relevant morphological and structural features. Figure 11 shows typical images of CdS quantum dots produced with chitosan (Figure 11A) and C-Pal (Figure 11B). It can be observed that both systems have nanoparticles with spherical morphology, with sizes of about 3–4 nm, and reasonably monodispersed, which is coherent with those values estimated by UV-vis spectroscopy measurements described in the previous section. The electron diffraction (ED) pattern of the QDs conjugates showed a lattice parameter comparable to the CdS wurtzite crystalline structure (sketch, inset of Figure 11B). Additionally, the chemical analysis by EDX spectroscopy (not shown) indicated Cd and S as the major elements, excluding the copper and carbon peaks from the TEM grid and Si from the detector. Thus, the TEM results have given supporting evidence that very small CdS quantum dots were produced and stabilized by the carbohydrate ligands.

#### 3.2.4. Dynamic Light Scattering (DLS) Analysis

The number-average size value of CdS_CHI and CdS_C-Pal were 28.4 ± 0.4 nm and 28.0 ± 0.1 nm respectively, with a polydisperisty index of 0.24 for both systems. These values correspond to a “hydrodynamic diameter” (H_D_) that is different of that primary semiconductor particles sizes calculated from UV-vis absorbance curve and TEM analysis (~3.5 nm). That could be attributed to the contributions of CdS QDs (“core”) and chitosan/chitosan derivative polymer (“organic shell”), including the influence of solvation layers, polyelectrolyte dispersion effects, and restrictions in bond and rotation angles, leading to larger sizes in colloidal water media than the “geometric” sizes estimated by TEM (“dry” morphological analysis) or UV-vis (energy of band gap absorption) techniques [52]. The similarity of the values, despite the presence of palmitoyl groups in C-Pal chitosan may be expected considering that both systems are in the same pH and the previously published results of H_D_ for chitosan derivatives [53]. Mochalova *et al*. [53] reported that an increase of 160% of the molecular mass of the chitosan by the incorporation of polyacrylamide polymer resulted in an increase of only 15% in the H_D_. That indicates a minor effect of the growth of side chain *via* amine group in the hydrodynamic diameter. In our case, an increase of 28% of the molar mass in palmitoyl chitosan derivative, in comparison with pure chitosan, is associated with the achieved degree of substitution of 20% (DA estimated through FTIR). In that sense, no relevant difference would be expected between CdS_CHI and CdS_Pal systems when an increase of 28% would result in slight changes in H_D_.

### 3.3. Biofunctionalized QDs for Potential Bioapplications

It can be summarized that novel biofunctionalized fluorescent systems were developed in this study for innumerous biomedical applications. Herein a potential application of the system based on the interaction of the QDs glycoconjugates with lipid molecules for health and nutrition purposes is briefly suggested. Owing to its distinct chemico-biological properties, chitosan and its derivatives may offer great potential in nutrition and pharmaceutical applications [46,54]. One relevant application is as a dietary antilipidemic supplement to be used to reduce obesity/overweight and to lower cholesterol. The lipid-binding efficiency of chitosans and derivatives, however, remains debatable. It can reduce the risk of cardiovascular diseases and has potent fat-binding capacity *in vitro* [4,55]. From the pharmaceutical perspective, the application of nanocarriers in drug formulation is one approach to reduce toxicity, efficiency and improve drug safety [56]. Consequently, it can be foreseen that the combination of fluorescent nanomaterials such as QDs with an amphiphilic polymer, such as chitosan and derivatives with hydrophobic and hydrophilic units within its structure have a very promising outlook. Ideally, the hydrophobic part of the amphiphilic biopolymer interacts with lipids and other low water soluble molecules, while the hydrophilic part provides water dispersibility and simultaneously chemical stability [57]. Thus, in this study, we synthesized luminescent CdS quantum dot functionalized by hydrophobically-modified chitosan with their physico-chemical characteristics thoroughly investigated. These water-soluble bioconjugates based on QDs and chitosan could be suggested as dietary supplements combining chemical affinity with lipids and their luminescent properties for tracking and/or detecting them in the digestive tract.

**Figure 12:**
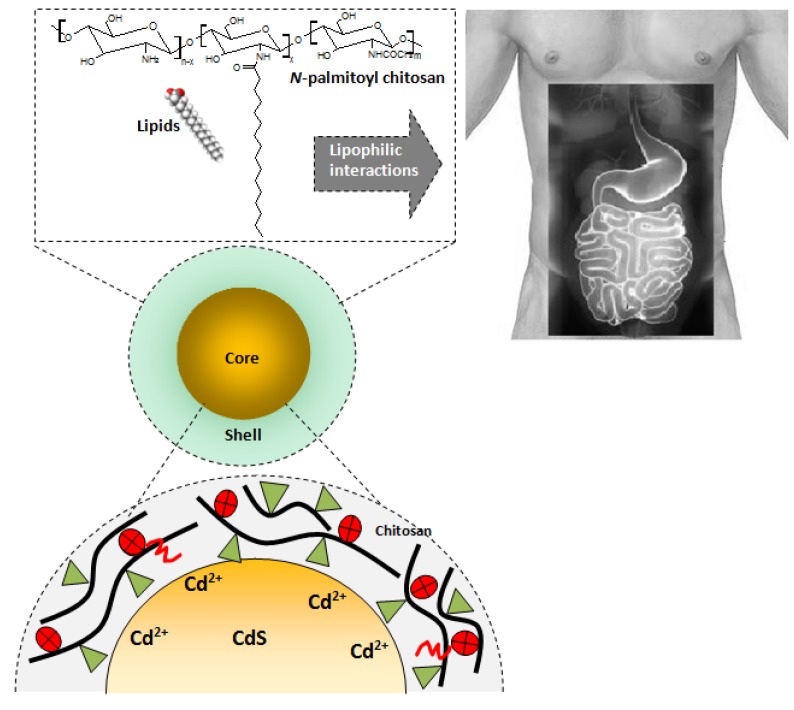
CdS QDs biofunctionalized with glycoconjugates designed for potential bioapplications involving lipophilic interactions in the digestive tract.

The idealized hypothetical bioapplication of the system developed in the present research is schematically depicted in Figure 12 (drawing not to scale). It should be highlighted that it is not recommended as a treatment of diseases or nutrition recipe, but only as a preliminary approach for utilizing the nanohybrids produced in this research. Undoubtedly, several studies must be carried out by researchers, nutritionists, scientists and other specialists for exploiting the large number of possibilities considering the *in vitro* and *in vivo* bioapplications of the novel fluorescent biofunctionalized systems introduced in this research.

## 4. Conclusions

In the present study, novel biofunctionalized CdS quantum dots conjugates were synthesized in aqueous media using chitosan and *N*-acylated chitosan as ligands via a single-step colloidal process. The chemical modification of chitosan by grafting palmitoyl groups was evidenced by FTIR spectroscopy to have significantly altered its hydrophobic/hydrophilic behavior as assessed by measurements of contact angle and the degree of swelling in phosphate buffer medium. Additionally, the chitosan and *N*-palmitoyl chitosan were effective as capping ligands for producing nano-hybrids with inorganic-organic “core-shell” structures exhibiting fluorescent photoactivity. Hence, based on the photoluminescent behavior and the physico-chemical properties of the biofunctionalized quantum dots, one may assume that these systems could be potentially exploited as fluorescent biomarkers in nanomedicine such as in lipid molecule sequestration, tracking, signaling, sensing and also as lipid-lowering drugs.
